# Supplementary material for: Modeling glioblastoma heterogeneity as a dynamic network of cell states
Source: Mol Syst Biol. 2021 Sep 16;17(9):e10105. doi: 10.15252/msb.202010105 (PMC8444284; doi:10.15252/msb.202010105)
Supplement: Supplementary file 6 — Source Data for Figure 5 [file MSB-17-e10105-s004.zip › Figure5A_sourcedata/GSEA_3071/nosigdb_stateVI.GseaPreranked.1622795169056/SUVA_NPC1_PMID_31327527.html]

Details for gene set SUVA\_NPC1\_PMID\_31327527[GSEA]

|  || Dataset | state63071 |
| Phenotype | NoPhenotypeAvailable |
| Upregulated in class | na\_neg |
| GeneSet | SUVA\_NPC1\_PMID\_31327527 |
| Enrichment Score (ES) | -0.7371238 |
| Normalized Enrichment Score (NES) | -2.7646365 |
| Nominal p-value | 0.0 |
| FDR q-value | 0.0 |
| FWER p-Value | 0.0 |
Table: GSEA Results Summary

  

Fig 1: Enrichment plot: SUVA\_NPC1\_PMID\_31327527      
 Profile of the Running ES Score & Positions of GeneSet Members on the Rank Ordered List

  

| PROBE | GENE SYMBOL | GENE\_TITLE | RANK IN GENE LIST | RANK METRIC SCORE | RUNNING ES | CORE ENRICHMENT || 1 | TSPAN13 |  |  | 1195 | 0.317 | -0.3189 | No |
| 2 | MEST |  |  | 1294 | 0.304 | -0.3347 | No |
| 3 | ABAT |  |  | 2438 | -0.365 | -0.6374 | No |
| 4 | GLCCI1 |  |  | 2521 | -0.383 | -0.6457 | No |
| 5 | STMN1 |  |  | 2646 | -0.411 | -0.6646 | No |
| 6 | MLLT11 |  |  | 2850 | -0.476 | -0.7030 | No |
| 7 | MARCKSL1 |  |  | 2928 | -0.506 | -0.7053 | No |
| 8 | TUBB3 |  |  | 3044 | -0.560 | -0.7161 | Yes |
| 9 | SOX4 |  |  | 3066 | -0.571 | -0.7005 | Yes |
| 10 | OLIG1 |  |  | 3126 | -0.601 | -0.6942 | Yes |
| 11 | PFN2 |  |  | 3215 | -0.685 | -0.6929 | Yes |
| 12 | TAGLN3 |  |  | 3373 | -0.842 | -0.7048 | Yes |
| 13 | BTG2 |  |  | 3377 | -0.857 | -0.6734 | Yes |
| 14 | ETV1 |  |  | 3378 | -0.857 | -0.6412 | Yes |
| 15 | HES6 |  |  | 3384 | -0.864 | -0.6102 | Yes |
| 16 | LRRN1 |  |  | 3420 | -0.942 | -0.5845 | Yes |
| 17 | HIP1 |  |  | 3438 | -0.981 | -0.5523 | Yes |
| 18 | SOX11 |  |  | 3443 | -0.989 | -0.5163 | Yes |
| 19 | TUBA1A |  |  | 3447 | -1.002 | -0.4795 | Yes |
| 20 | CHD7 |  |  | 3454 | -1.017 | -0.4430 | Yes |
| 21 | DLL3 |  |  | 3460 | -1.030 | -0.4057 | Yes |
| 22 | TCF12 |  |  | 3494 | -1.100 | -0.3735 | Yes |
| 23 | MAP2 |  |  | 3597 | -1.763 | -0.3356 | Yes |
| 24 | ASCL1 |  |  | 3604 | -1.822 | -0.2688 | Yes |
| 25 | BEX1 |  |  | 3608 | -1.883 | -0.1989 | Yes |
| 26 | FXYD6 |  |  | 3628 | -2.707 | -0.1025 | Yes |
| 27 | DCX |  |  | 3630 | -2.797 | 0.0022 | Yes |
Table: GSEA details [plain text format]

  

Fig 2: SUVA\_NPC1\_PMID\_31327527: Random ES distribution      
 Gene set null distribution of ES for **SUVA\_NPC1\_PMID\_31327527**
